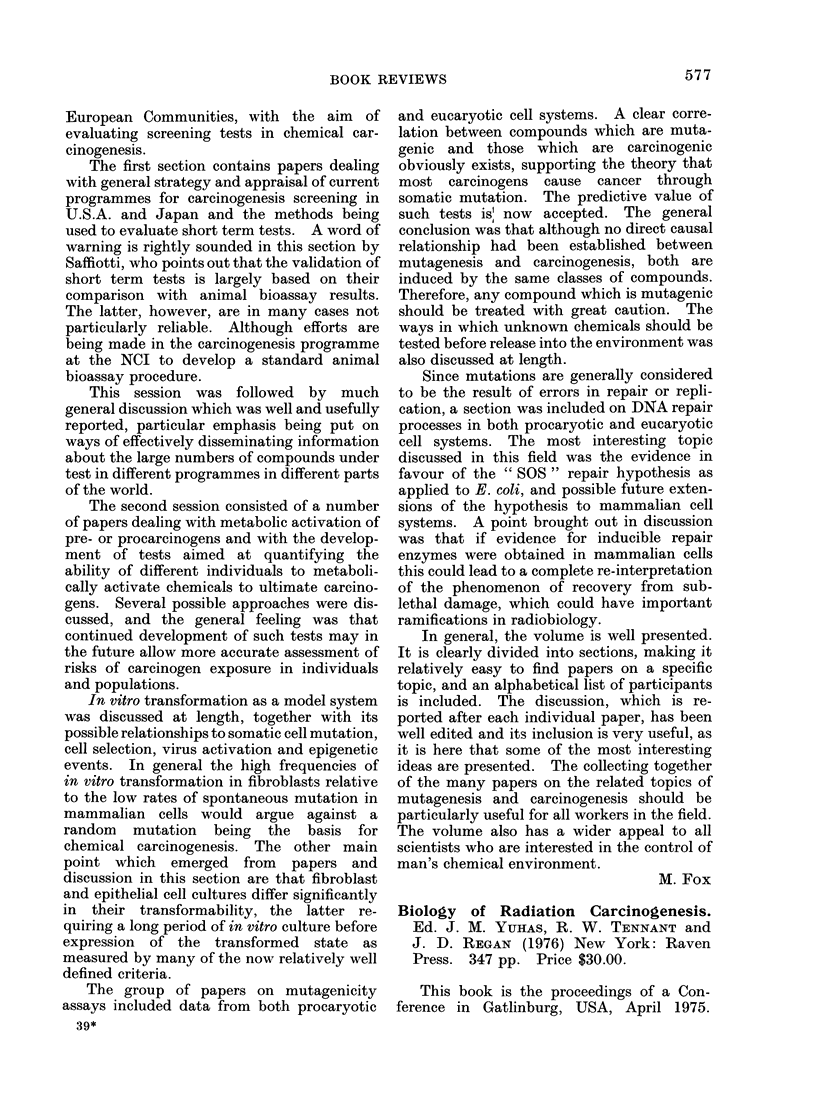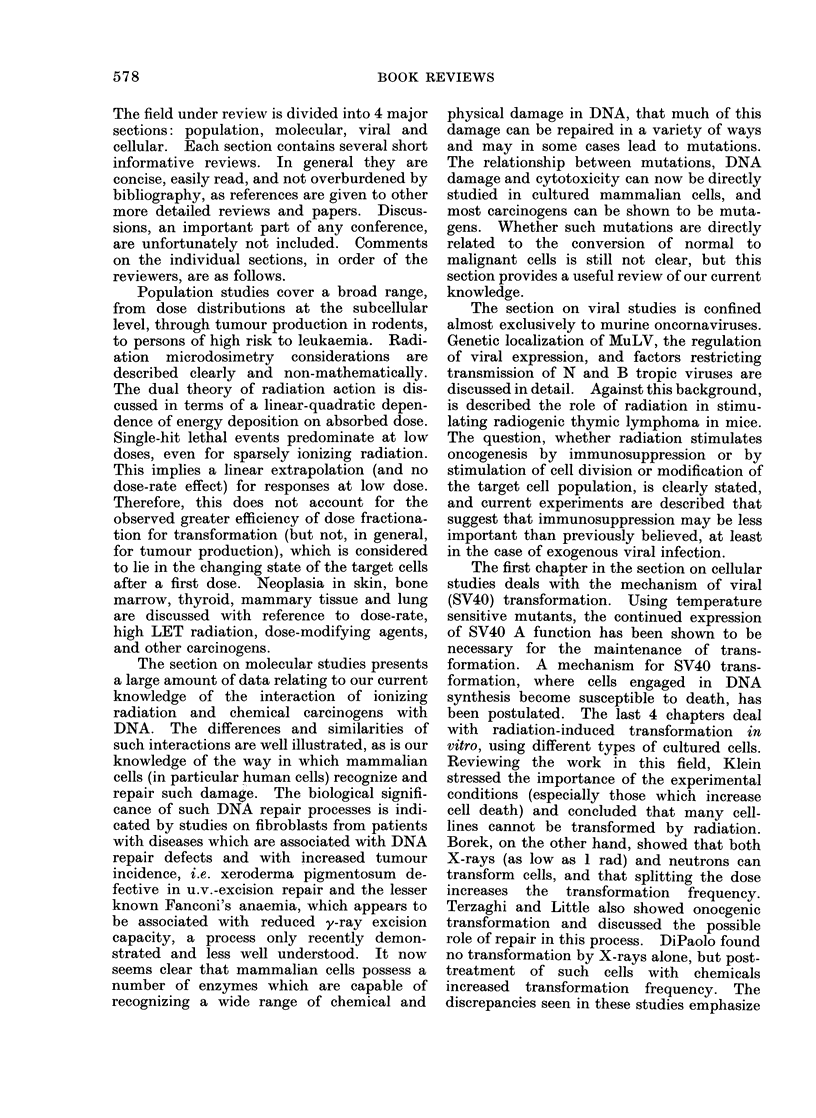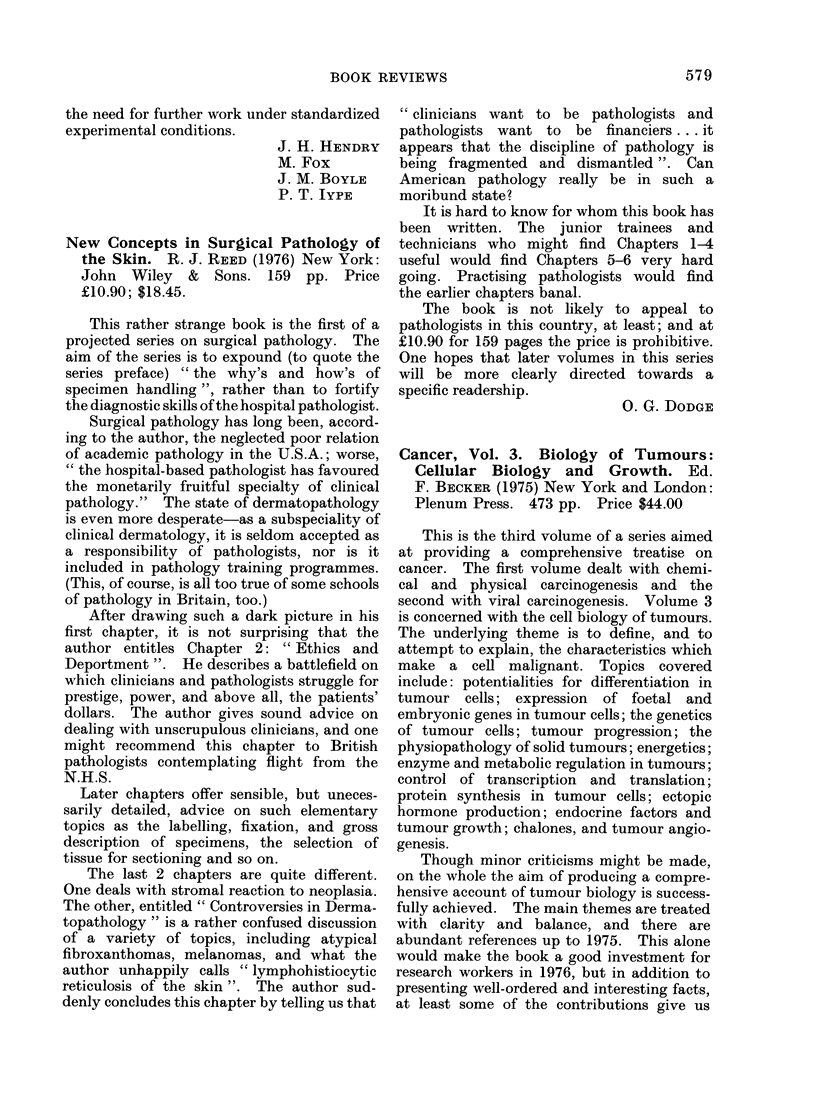# Biology of Radiation Carcinogenesis

**Published:** 1976-11

**Authors:** J. H. Hendry, M. Fox, J. M. Boyle, P. T. Iype


M. Fox

**Biology of Radiation Carcinogenesis.** Ed. J. M. Yuhas, R. W. Tennant and J. D. Regan (1976) New York: Raven Press. 347 pp. Price $30.00.

This book is the proceedings of a Conference in Gatlinburg, USA, April 1975.

The field under review is divided into 4 major sections: population, molecular, viral and cellular. Each section contains several short informative reviews. In general they are concise, easily read, and not overburdened by bibliography, as references are given to other more detailed reviews and papers. Discussions, an important part of any conference, are unfortunately not included. Comments on the individual sections, in order of the reviewers, are as follows.

Population studies cover a broad range, from dose distributions at the subcellular level, through tumour production in rodents, to persons of high risk to leukaemia. Radiation microdosimetry considerations are described clearly and non-mathematically. The dual theory of radiation action is discussed in terms of a linear-quadratic dependence of energy deposition on absorbed dose. Single-hit lethal events predominate at low doses, even for sparsely ionizing radiation. This implies a linear extrapolation (and no dose-rate effect) for responses at low dose. Therefore, this does not account for the observed greater efficiency of dose fractionation for transformation (but not, in general, for tumour production), which is considered to lie in the changing state of the target cells after a first dose. Neoplasia in skin, bone marrow, thyroid, mammary tissue and lung are discussed with reference to dose-rate, high LET radiation, dose-modifying agents, and other carcinogens.

The section on molecular studies presents a large amount of data relating to our current knowledge of the interaction of ionizing radiation and chemical carcinogens with DNA. The differences and similarities of such interactions are well illustrated, as is our knowledge of the way in which mammalian cells (in particular human cells) recognize and repair such damage. The biological significance of such DNA repair processes is indicated by studies on fibroblasts from patients with diseases which are associated with DNA repair defects and with increased tumour incidence, *i.e.* xeroderma pigmentosum defective in u.v.-excision repair and the lesser known Fanconi's anaemia, which appears to be associated with reduced $\gamma$-ray excision capacity, a process only recently demonstrated and less well understood. It now seems clear that mammalian cells possess a number of enzymes which are capable of recognizing a wide range of chemical and physical damage in DNA, that much of this damage can be repaired in a variety of ways and may in some cases lead to mutations. The relationship between mutations, DNA damage and cytotoxicity can now be directly studied in cultured mammalian cells, and most carcinogens can be shown to be mutagens. Whether such mutations are directly related to the conversion of normal to malignant cells is still not clear, but this section provides a useful review of our current knowledge.

The section on viral studies is confined almost exclusively to murine oncornaviruses. Genetic localization of MuLV, the regulation of viral expression, and factors restricting transmission of N and B tropic viruses are discussed in detail. Against this background, is described the role of radiation in stimulating radiogenic thymic lymphoma in mice. The question, whether radiation stimulates oncogenesis by immunosuppression or by stimulation of cell division or modification of the target cell population, is clearly stated, and current experiments are described that suggest that immunosuppression may be less important than previously believed, at least in the case of exogenous viral infection.

The first chapter in the section on cellular studies deals with the mechanism of viral (SV40) transformation. Using temperature sensitive mutants, the continued expression of SV40 A function has been shown to be necessary for the maintenance of transformation. A mechanism for SV40 transformation, where cells engaged in DNA synthesis become susceptible to death, has been postulated. The last 4 chapters deal with radiation-induced transformation *in vitro*, using different types of cultured cells. Reviewing the work in this field, Klein stressed the importance of the experimental conditions (especially those which increase cell death) and concluded that many cell-lines cannot be transformed by radiation. Borek, on the other hand, showed that both X-rays (as low as 1 rad) and neutrons can transform cells, and that splitting the dose increases the transformation frequency. Terzaghi and Little also showed onocgenic transformation and discussed the possible role of repair in this process. DiPaolo found no transformation by X-rays alone, but post-treatment of such cells with chemicals increased transformation frequency. The discrepancies seen in these studies emphasize the need for further work under standardized experimental conditions.

J. H. HENDRY
M. FOX
J. M. BOYLE
P. T. IYPE